# LncRNA CCAT1 is overexpressed in tuberculosis patients and predicts their survival

**DOI:** 10.1002/iid3.565

**Published:** 2021-11-30

**Authors:** Taosheng Ye, Jiaohong Zhang, Xuan Zeng, Yuxiang Xu, Jinpei Li

**Affiliations:** ^1^ Department of Respiratory Endoscopy The Third People's Hospital of Shenzhen Shenzhen Guangdong China

**Keywords:** CCAT1, IL‐10, survival, tuberculosis

## Abstract

**Introduction:**

LncRNA CCAT1 promotes inflammatory responses, which contribute to tuberculosis. Therefore, CCAT1 may participate in tuberculosis. Therefore, we analyzed the involvement of CCAT1 in tuberculosis.

**Methods:**

Plasma samples were donated by a total of 200 patients with newly developed tuberculosis (N‐TB), 102 patients with recurrent tuberculosis (R‐TB), and 102 healthy controls on the day of admission. Plasma samples were also collected from N‐TB and R‐TB patients every month after the initiation of treatment for a total of 6 months. CCAT1 expression in these samples was detected by quantitative reverse transcription polymerase chain reaction. Levels of IFN‐γ, IL‐1β, iNOS, TNF‐α, and IL‐10 in plasma were determined by enzyme‐linked immunosorbent assay. N‐TB and R‐TB patients were monitored for 2 months to analyze their survival.

**Results:**

On the day of admission, the highest levels of CCAT1, IFN‐γ, IL‐1β, iNOS, and TNF‐α were detected in N‐TB patients, followed by R‐TB patients and controls, while the lowest levels of plasma IL‐10 were detected in N‐TB patients, followed by R‐TB patients and controls. Across R‐TB and N‐TB patients, CCAT1 was inversely correlated with IL‐10 but not closely correlated with other inflammatory factors. During the treatment, plasma CCAT1 levels decreased in both N‐TB and R‐TB patients. High CCAT1 levels were closely correlated with high mortality rates of both N‐TB and R‐TB patients.

**Conclusion:**

CCAT1 is overexpressed in tuberculosis patients and predicts their survival. Its function in tuberculosis may be related to IL‐10.

## 1 INTRODUCTION

As a common clinical disorder caused by the infection of *Mycobacterium tuberculosis*, tuberculosis (TB) mainly affects the lungs, while damages of other organs caused by TB are also observed.^1^, ^2^ The incidence of TB varies greatly across countries. It is estimated that TB only affects about 2.7 out of 100,000 people in the United States, while the incidence rate is as high as 100 out of 100,000 people in many places of China.^3^, ^4^ In 2015, about 10.4 million new TB cases were diagnosed. Therefore, TB remains a heavy burden of public health.^5^ TB patients are usually treated with antibiotics, such as isoniazid and rifampicin.^6^, ^7^ However, TB‐caused deaths, which are mainly related to infections of multidrug‐resistant strains and delayed treatment, are still common.^8^

*M. tuberculosis* can evade the host immune response, causing inflammation in the lungs.^9^, ^10^ Currently, inflammation in TB is irresolvable. Therefore, inhibiting inflammation response is still a key for the treatment of TB.^9^, ^10^ However, the mechanisms that mediate the inflammation in TB have not been fully elucidated.^9^, ^10^ Previous studies have characterized a considerable number of lncRNAs with altered expression in TB.^11^, ^12^ Some lncRNAs participate in TB‐related inflammation to affect TB progression.^13^, ^14^ However, the function of most lncRNAs in TB is unclear. LncRNA CCAT1 promotes inflammatory responses,^15^ which contribute to tuberculosis, suggesting that CCAT1 may participate in TB. Therefore, we analyzed the involvement of CCAT1 in TB.

## 2 MATERIALS AND METHODS

### 2.1 Patients

The present study enrolled a total of 200 patients with newly developed tuberculosis (N‐TB; 110 males and 90 females; 38.1 ± 8.8 years), 102 patients with recurrent tuberculosis (R‐TB; 55 males and 47 females; 37.7 ± 8.9 years), and 102 healthy controls (55 males and 47 females; 37.8 ± 9.0 years). All these patients were admitted to The Third people's Hospital of Shenzhen from March 2017 to March 2021. No significant differences in age and gender were observed among these three groups. Tuberculosis patients were diagnosed through the detection of bacterial infection, clinical manifestations, and imaging analysis. All patients were Mtb type. All healthy controls received routine physiological examinations and had normal physiological functions. Patients and controls were excluded if they had other infections, such as fungus and viruses, metabolic diseases, cancers, and other severe diseases. Pregnant women were excluded. Ethics approval was obtained from our hospital. Informed consent was provided by all participants.

### 2.2 Plasma samples, treatment, and survival analysis

All patients were treated with soniazid INH combined with rifampin, pyrazinamide, and ethambutol with various dosages. On the day of admission, peripheral blood samples (5 ml) were extracted from all participants under fasting conditions. The same amount of peripheral blood was also extracted from both N‐TB and R‐TB patients every month for a total of 6 months after the initiation of therapy. Blood samples were used to prepare plasma samples. After the admission of patients, a 2‐month follow‐up study was performed to monitor patients' survival. The 200 N‐TB and 102 R‐TB were divided into high and low CCAT1 levels groups with the median CCAT1 level as the cut‐off. Survival curves were plotted and compared by logrank test.

### 2.3 RNA sample preparations and analysis

Total RNAs were isolated using EZ Tissue/Cell Total RNA Miniprep Kit from 5 × 10^6^ in vitro cultured cells or 30 mg samples from patients. After cell lysis, RNA samples were purified using columns and treated with DNase included in the kit to remove DNA contamination. Before the subsequent assays, RNA integrity and concentrations were analyzed using Bioanalyzer. Only samples with concentration higher than 2000 ng/μl and RIN value higher than 8.5 were used in the preparation of cDNAs.

### 2.4 Gene expression analysis with reverse transcriptions and quantitative polymerase chain reactions

cDNA samples were prepared by reverse transcriptions (RTs) using GoScript™ Reverse Transcriptase kit (Promega). In brief, 5 µg total RNA samples were mixed with 0.5 µl primer and nuclease‐free water in a 5 µl system, incubated at 70°C for 5 min and placed on ice for at least 5 min. After that, the mixture was combined with 15 µl solution containing 4.0 µl GoScript™ 5X Reaction Buffer, 2.0 µl MgCl_2_, 1.0 µl PCR Nucleotide Mix 2, 20 U recombinant RNasin® Ribonuclease Inhibitor, and 1.0 µl GoScript™ Reverse Transcriptase and incubated at 25°C for 5 min, 42°C for 30 min, and 70°C for 15 min to synthesize cDNA samples. CCAT1 expression was determined using qPCRs using cDNA templates with 18S rRNA as the internal control. *C*_t_ values were normalized to the internal controls using the $2^{‐\Delta \Delta C_{t}}$ method.

### 2.5 Enzyme‐linked immunosorbent assay

Plasma levels of IFN‐γ, IL‐1β, iNOS, TNF‐α, and IL‐10 were determined using Human IFN‐γ ELISA Kit (ab46025; Abcam), Human IL‐1β ELISA Kit (ab46052; Abcam), Human iNOS ELISA Kit (ab253217; Abcam), Human TNF‐α ELISA Kit (ab181421; Abcam), and Human IL‐10 ELISA Kit (ab46034; Abcam), respectively, following the manufacturer's instructions.

### 2.6 Statistical analysis

Differences among more than two groups were analyzed using analysis of variance Tukey's test. The 200 N‐TB and 102 R‐TB were divided into high and low CCAT1 levels groups with the median CCAT1 level on the day of admission as the cut‐off value. Survival curves were plotted and compared by logrank test. *p* < 0.05 was statistically significant.

## 3 RESULTS

### 3.1 Plasma levels of CCAT1, IFN‐γ, IL‐1β, iNOS, TNF‐α, and IL10 in patients and controls

**Figure 1:**
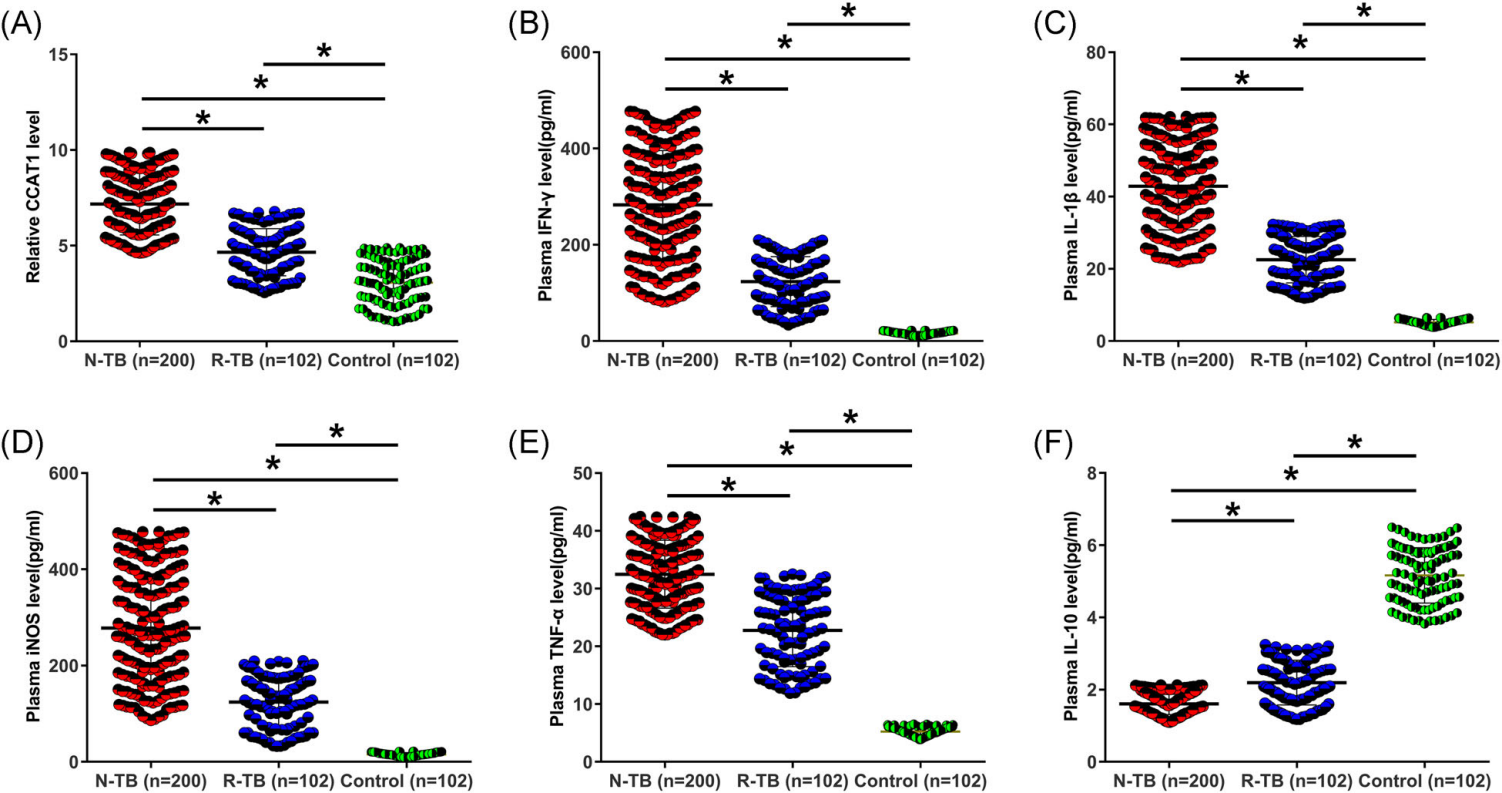
Plasma levels of CCAT1, IFN‐γ, IL‐1β, iNOS, TNF‐α, and IL10 in patients and controls. Plasma samples from 200 newly developed tuberculosis (N‐TB) patients, 102 recurrent tuberculosis (R‐TB) patients, and 102 controls were subjected to both quantitative reverse transcription polymerase chain reaction and enzyme‐linked immunosorbent assay to determine plasma levels of CCAT1 (A), IFN‐γ (B), IL‐1β (C), iNOS (D), TNF‐α (E), and IL‐10 (F). *p < 0.05

Plasma samples from 200 N‐TB patients, 102 R‐TB patients, and 102 controls were subjected to both quantitative RT polymerase chain reaction (RT‐qPCR) and enzyme‐linked immunosorbent assay to determine plasma levels of CCAT1, IFN‐γ, IL‐1β, iNOS, TNF‐α, and IL‐10. On the day of admission, the highest levels of CCAT1 (Figure 1A, *p* < 0.05), IFN‐γ (Figure 1B, *p* < 0.05), IL‐1β (Figure 1C, *p* < 0.05), iNOS (Figure 1D, *p* < 0.05), and TNF‐α (Figure 1E, *p* < 0.05) were detected in N‐TB patients, followed by R‐TB patients and controls, while the lowest levels of plasma IL‐10 were detected in N‐TB patients, followed by R‐TB patients and controls (Figure 1F, *p* < 0.05).

### 3.2 CCAT1 was inversely correlated with IL‐10 but not closely correlated with other inflammatory factors

**Figure 2:**
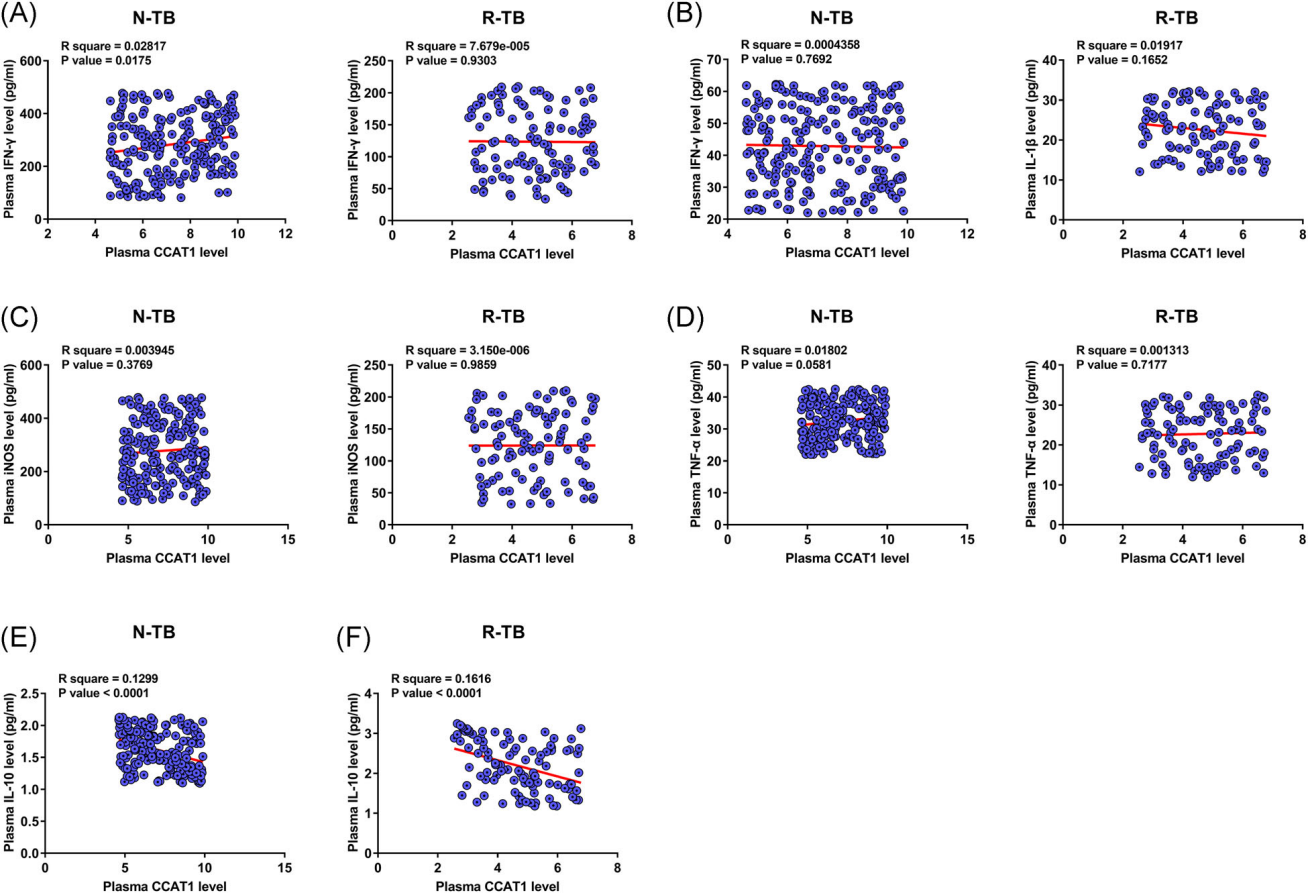
CCAT1 was inversely correlated with IL‐10 but not closely correlated with other inflammatory factors. Pearson's correlation coefficient was performed to determine the correlations between CCAT1 and IFN‐γ (A), IL‐1β (B), iNOS (C), TNF‐α (D), and IL‐10 (E) across both newly developed tuberculosis (N‐TB) and recurrent tuberculosis (R‐TB) plasma samples

Pearson's correlation coefficient was performed to determine the correlations of CCAT1 with IFN‐γ (Figure 2A), IL‐1β (Figure 2B), iNOS (Figure 2C), TNF‐α (Figure 2D), and IL‐10 (Figure 2E) across both N‐TB and R‐TB plasma samples. CCAT1 was inversely correlated with IL‐10 (Figure 2F) across R‐TB and N‐TB patients but not closely correlated with other inflammatory factors.

### 3.3 Plasma CCAT1 levels decreased in both N‐TB and R‐TB patients during treatment

**Figure 3:**
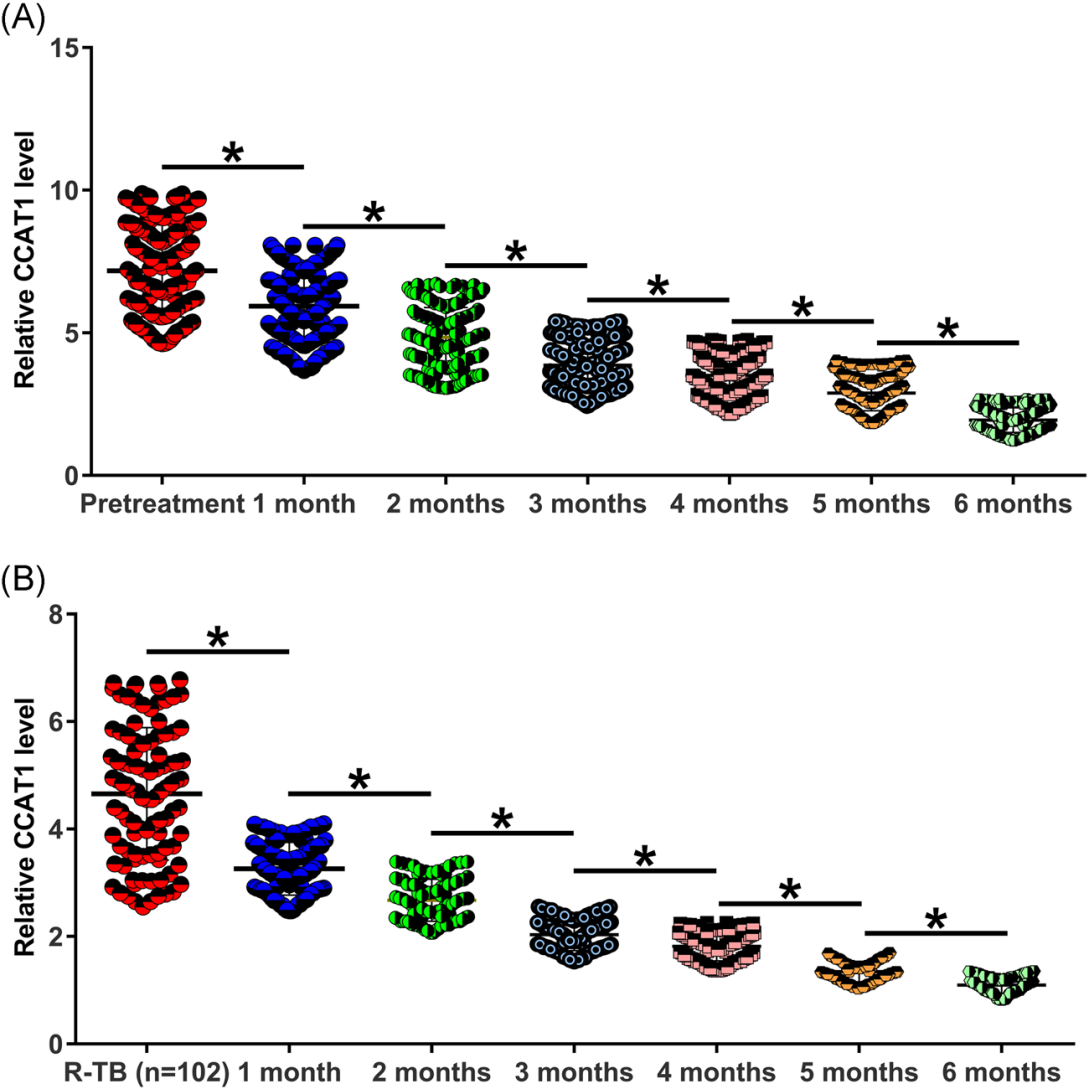
Plasma CCAT1 levels decreased in both newly developed tuberculosis (N‐TB) and recurrent tuberculosis (R‐TB) patients during treatment. CCAT1 levels in plasm samples from both N‐TB (A) and R‐TB (B) patients were measured every month for a total of 6 months through RNA isolation and quantitative reverse transcription polymerase chain reactions analysis. *p < 0.05

Plasma CCAT1 levels in both N‐TB and R‐TB patients were measured every month for a total of 6 months. Because some patients died during the follow‐up, 200, 193, 188, 185, 183, 181, and 178 patients were included in N‐TB group at pretreatment, and 1, 2, 3, 4, 5, and 6 months after the initiation of treatment, respectively. Similarly, 102, 99, 97, 95, 93, 91, and 90 patients were included in the R‐TB group at pretreatment and 1, 2, 3, 4, 5, and 6 months after the initiation of treatment, respectively. RNA isolation and RT‐qPCRs analysis showed that plasma CCAT1 levels decreased in both N‐TB (Figure 3A, *p* < 0.05) and R‐TB (Figure 3B, *p* < 0.05) patients during the treatment.

### 3.4 High CCAT1 levels were closely correlated with high mortality rates of both N‐TB and R‐TB patients

**Figure 4:**
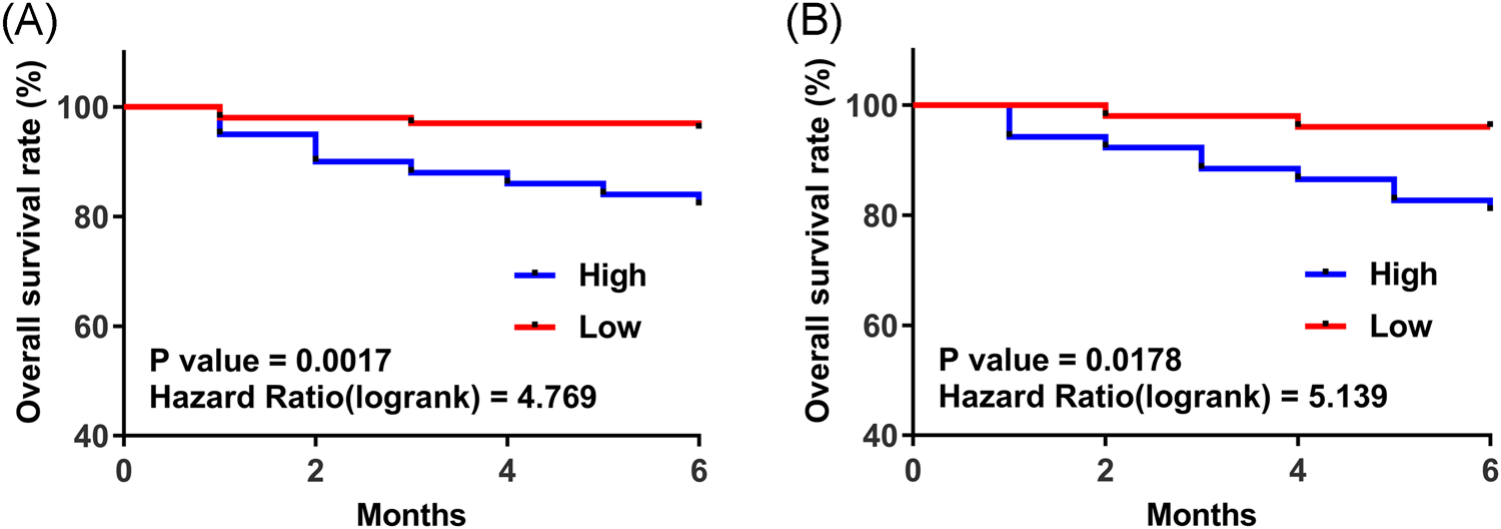
High CCAT1 levels were closely correlated with a high mortality rate for both newly developed tuberculosis (N‐TB) and recurrent tuberculosis (R‐TB) patients. A 2‐month follow‐up study was performed to analyze the prognostic value of CCAT1 for both N‐TB (A) and R‐TB (B). The 200 N‐TB and 102 R‐TB patients were divided into high and low CCAT1 level groups with the median CCAT1 level as the cut‐off values. Survival curves were plotted and compared by logrank test.

A 2‐month follow‐up study was performed to analyze the prognostic value of CCAT1 for both N‐TB and R‐TB. The 200 N‐TB and 102 R‐TB were divided into high and low CCAT1 level groups with the median CCAT1 levels on the day of admission as the cut‐off values. In the N‐TB group, 22 deaths were observed, and the causes of death were bacterial infection (*n* = 6), cerebral vascular diseases (*n* = 4), hepatic failure (4), renal failure (3), cardiovascular diseases (*n* = 3), and autoimmune (*n* = 2). In the R‐TB group, 10 deaths were observed, and the causes of death were bacterial infection (*n* = 3), cerebral vascular diseases (*n* = 2), hepatic failure (2), cardiovascular diseases (*n* = 2), and AIDS (*n* = 1). Survival curves were plotted and compared by logrank test. No significant differences in causes of death were observed between high and low CCAT1 level groups in both N‐TB and R‐TB (data not shown). Association analysis also showed that CCAT1 was not closely associated with age, gender, smoking and drinking habit, infections, and other clinical factors in both N‐TB and R‐TB patients (data not shown). It was observed that high CCAT1 levels were closely correlated with the high mortality rate of both N‐TB (Figure 4A) and R‐TB (Figure 4B) patients.

## 4 DISCUSSION

The present study explored the role of CCAT1 in TB. We showed that CCAT1 is overexpressed in TB patients and predicts survival. Its function in tuberculosis may be related to IL‐10.

A recent study reported CCAT1 as a proinflammatory factor in inflammatory bowel disease. It was observed that CCAT1 expression was altered in inflammatory bowel disease, and CCAT1 overexpression downregulates miR‐185‐3p to destroy the intestinal barrier, thereby promoting disease progression.^15^ TB is also an inflammatory disease. In the present study, we observed CCAT1 upregulation in both N‐TB and R‐TB patients. Interestingly, higher plasma CCAT1 levels were observed in N‐TB patients than in R‐TB patients. TB recurrence after cure is common.^1^, ^2^ The differential expression of CCAT1 in N‐TB and R‐TB patients may be caused by existing antibodies in R‐TB patients, which may suppress CCAT1 expression. However, there is no evidence to support the involvement of antibodies in CCAT1 expression. Future studies are needed to explore this possibility. Plasma CCAT1 levels decreased continuously with the treatment of TB. Therefore, CCAT1 expression may reflect the treatment outcomes of TB. Interestingly, on Month 6 (Figure 3), CCAT1 expression levels in both N‐RB and R‐TB groups were even lower than that in the control group (Figure 1A). In this study, all patients were treated with soniazid INH combined with rifampin, pyrazinamide, and ethambutol. These chemicals may have inhibitory effects on CCAT1.

It is estimated that more than 10% of TB patients will die of TB within 2 months of admission.^8^ In this study, we showed that high plasma CCAT1 levels on the day of admission were closely correlated with the worse survival of both N‐TB and R‐TB patients. Therefore, measuring CCAT1 expression levels on the day of admission may help to identify patients with a high mortality risk, thereby assisting the design of treatment strategies to improve patients' survival.

The progression of TB is closely correlated with various inflammatory factors.^16^, ^17^, ^18^ Among them, TNF‐α and IL‐10 are characterized as major factors influencing this disease.^16^ In this study, we observed altered expression of IFN‐γ, IL‐1β, iNOS, TNF‐α, and IL‐10 in TB, while CCAT1 was only correlated with IL‐10 but not other inflammatory factors. Therefore, CCAT1 may participate in TB mainly by interacting with IL‐10. However, there are no previous studies on the interaction between CCAT1 and IL‐10. Therefore, this speculation needs to be further validated.

In conclusion, CCAT1 is overexpressed in TB and may participate in TB by regulating IL‐10. Moreover, CCAT1 expression may predict the treatment outcomes and survival of TB patients.

## CONFLICT OF INTERESTS

The authors declare that there are no conflict of interests.

## ETHICS STATEMENT

All patients signed written informed consent. All procedures were approved by the Ethics Committee of The Third People's Hospital of Shenzhen and operated in keeping with the standards set out in the Announcement of Helsinki and Laboratory Guidelines of Research in China.

## AUTHOR CONTRIBUTIONS

*Study concepts, literature research, clinical studies, data analysis, experimental studies, manuscript writing and review*: Taosheng Ye. *Study design, literature research, experimental studies and manuscript editing*: Taosheng Ye and Jiaohong Zhang. *Definition of intellectual content, clinical studies, data acquisition and statistical analysis*: Xuan Zeng. *Data acquisition, manuscript preparation, and data analysis*: Yuxiang Xu. *Data acquisition and statistical analysis*: Jinpei Li. All authors have read and approved the submission of the manuscript.

## Data Availability

The data that support the findings of this study are available on request from the corresponding author. The data are not publicly available due to their containing information that could compromise the privacy of research participants.
